# On the Simultaneous Development of Variola and Vaccinia

**Published:** 1849-09

**Authors:** 


*On the Simultaneous Development of Variola and Vaccinia.* By MM. Herard and Bousquet.—M. Herard having had, at the Children's Hospital at Paris (where children are so wantonly exposed to the danger of contracting small pox prior to their vaccination), the opportunity of observing eighteen cases of the co-existence of the two diseases, he is desirous of expressing his opinion upon the disputed question as to the degree of influence they exert upon each other.

Of these eighteen children (from twenty months to four years old) seven died; all these being, however, cachectic, or exhausted by prior disease. In them, the small-pox was rarely confluent, but it was irregular and ill developed. In the eleven cases which recovered, the eruption was discrete, rapid in its course, and unaccompanied by suppuration of the pustules, or tumefaction of the surrounding skin. The fever was slight, and the convalescence rapid. During this benign progress of these cases, the disease more than once proved very fatal to non-vaccinated children. Had the vaccine acted injuriously in the seven children who died? MM. Rilliet, Barthez, and Legendre answer this question in the affirmative, believing the vaccination hastens the evolution of the variola, and increases the debility—opinions with which M. Herard does not agree. He also, in the eleven successful cases, observed no difference produced in the mode of the development of the vaccine vesicle; but in the seven children who died from various complications the vaccine was in a very languishing condition. The following are the general conclusions arrived at:

1. When the two eruptions are developed at the same time in a healthy child, the variola is advantageously modified. Its progress is more rapid, and the eruption is more discrete—taking on the character of varioloid.

2. Although vaccination is far from exerting the same effect, and especially in hospitals, on very young and diseased children, being innocent, it should be practiced. The danger in these cases is attributable to the small-pox not to the vaccination, and is produced by complications and bad hygienic conditions.

3. It is not correct to say that the two eruptions exert a reciprocal modification. That which has the priority of invasion (not of infection) influences the other, but is not influenced by it; and as, in almost all cases, the vaccine, to become developed, must precede the variola, we may generally state that, in the cases where the diseases exist simultaneously, the vaccinia undergoes no modification. —*L'Union Médicale.*

M. Bousquet states that an attentive examination of the voluminous correspondence of the Académie reveals the greatest discrepancy of opinion upon this subject. For his own part, he denies that vaccinia modifies the progress of small-pox advantageously; and considers it a false deduction to suppose that, because vaccinia prevents variola, it must restrain its advantages when the two diseases meet together. According to MM. Rayer and Clerault's statistics, of one hundred and eleven cases of simultaneous existence of the two diseases, twelve died, and M. Legendre states that nine of fifty-six died; but small-pox, unaided by vaccination, is not more fatal than this. Of M. Herard's eighteen cases, seven died, and there is no reason to agree with him that the remaining eleven were milder because of the vaccinia. MM. Rilliet and Barthez declare that in very young infants it increases danger; but M. Bousquet agrees with neither party. Those who maintain that small-pox is rendered more discrete by vaccination, take only the successful cases, forgetting that, in the natural small-pox, the discrete exceed the confluent cases in the proportion of ten to one.

M. Bousquet therefore denies that the two eruptions exert any reciprocal reaction; and the nearer they appear together, the more independent are they of each other. Suppose, e. g., that the two could appear at the same hour; then each would pursue its ordinary course, just as if the other were not present. But supposing the one eruption appears after the other, all will depend upon the space of time separating them. If this be only some hours, or even two or three days, all passes on as just stated. The case is different, when one of these eruptions is greatly in advance of the other. If this is not to such an extent as to exclude, the eruptions progress together, but not in parallel. The most advanced always keeps its advantage, and finishes at its ordinary epoch, without having undergone any change in form or duration. The other follows it at a distance; but after the variolous capacity of the subject has become exhausted by the first, the second dies away. In these influences there is nothing direct, active, or special; they are the consequences of the faculty possessed by the eruptions of supplying or substituting each other. The vaccinia does not arrest the variola, but it is the variola that stops short in the face of the vaccinia; and, conversely, variola does not cut short the course of vaccinia, but this last interrupts its own course in presence of the variola. It is the right of precedence; and the more widely the two eruptions are separated, the more readily do they exclude each other; while the nearer they are together, the more independently do they proceed. Considered in themselves, the vaccine and variolous virus are so little capable of destroying each other's energy, that if they are mixed together, and inoculation performed with the mixture, two perfectly distinct eruptions are produced. Considered as regards their effects, we cannot say that vaccinia *cures* variola, or even, rigorously speaking, that it *prevents* it. It takes its place, stands in its stead, and is neither more nor less than a substitution. Thus, so far from explaining the operation of vaccinia by the supposed opposition it offers to variola, we would rather do so by the analogy and reciprocal action of the two diseases.—*Bulletin de Thérapeutique.*

